# Mortalin stabilizes CD151-depedent tetraspanin-enriched microdomains and implicates in the progression of hepatocellular carcinoma

**DOI:** 10.7150/jca.36301

**Published:** 2019-10-15

**Authors:** Li-Xin Liu, Jia-Cheng Lu, Hai-Ying Zeng, Jia-Bin Cai, Peng-Fei Zhang, Xiao-Jun Guo, Xiao-Yong Huang, Rui-Zhao Dong, Chi Zhang, Qiang Kang, Hao Zou, Xin-Yu Zhang, Lu Zhang, Xiao-Wen Zhang, Ai-Wu Ke, Guo-Ming Shi

**Affiliations:** 1 Department of Liver Surgery and Liver transplantation of Liver Cancer Institute & Zhongshan Hospital, Fudan University; Key Laboratory of Carcinogenesis and Cancer Invasion (Fudan University), Ministry of Education, Shanghai 200032, China; 2 Department of Hepatobiliary Surgery, Second Affiliated Hospital of Kunming Medical University, Kunming 650101, China; 3 Department of Pathology, Zhongshan Hospital, Fudan University, Shanghai 200032, P.R. China

**Keywords:** hepatocellular carcinoma, CD151, mortalin, tetraspanin-enriched microdomains, invasion

## Abstract

**Background:** Our previous studies showed that tetraspanin CD151 was implicated in the progression of hepatocellular carcinoma (HCC), mainly depending on the formation of functional complexes with molecular partners, including Mortalin. In this study, we investigate the role of mortalin in CD151-depedent progression of HCCs.

**Methods:** Immunofluorescent staining, western blot and quantitative real-time polymerase chain reaction (qRT-PCR) were used to investigate the expression and location of CD151 and Mortalin in four HCC cell lines with different metastatic ability. The relationship between Mortalin and CD151 was investigated in HCCLM3 cells using co-immunoprecipitation. CD151 or Mortalin expression in HCC cells were modified by transfection technology. Wound-healing assay and Transwell assay were used to assay the role of CD151 and Mortalin in cell migration and invasion. The expression and prognostic implication of CD151 and Mortalin in 187 cases of HCCs were analyzed.

**Results:** Expression of Mortalin in HCC cells was positive related to their metastatic ability and its tendency was in line with the expression of CD151. Immunofluorescent staining showed that Mortalin was located in cytoplasm, while positive staining for CD151 was observed in cytoplasm and membrane of HCC cells. co-IP revealed that Mortalin formed a complex with CD151. Down-regulation of Mortalin induced a moderate decreased CD151 protein, but not CD151 mRNA, while inhibition of CD151 did not influence the expression of Mortalin at the level of both protein and mRNA. Interference of Mortalin significantly inhibited the invasion and migration of HCC cells with high CD151 expression and partially restored the invasion and migration of HCC cells induced by CD151 over-expression. Clinically, high Mortalin expression correlated with malignant phenotype of HCC, such as microvascular invasion (*p*=0.017) and tumor diameter (*p*=0.001). HCC patients expressing high Mortalin were tend to have higher expression of CD151. HCC patients expressing high level of CD151 showed the poorer prognosis in a Mortalin-dependent manner.

**Conclusions:** Mortalin maybe stabilize of the structure of CD151-dependent tetraspanin-enriched microdomains and implicate in the progression of HCC.

## Introduction

As one of the most important member of the transmembrane 4 superfamily (TM4SF), it is well documented that tetraspanin CD151 is expressed in a wide range of normal cells such as endothelial cells and platelets, and involves in several physiological processes, such cell adhesion, motility, activation and proliferation 1. Recent evidences have recorded that CD151 frequently overexpresses in malignant tumor tissues, including hepatocellular carcinoma (HCC) 2, gallbladder carcinoma 3, breast cancer 4 and ovarian cancer 5, and acts as a “driver” in tumor progression through formation of tetraspanin CD151-enriched microdomains 2. Moreover, it is crucial for the function of CD151 to keep the structural stability of tetraspanin-enriched microdomains (TEM) 6. Our serial studies showed that CD151 involved in several pathological processes, including invasive ability, tumor neoangiogenesis and epithelial-mesenchymal transition (EMT), and then accelerated the invasion and metastasis in HCC 2, 7, 8. Based on the combination co-immunoprecipitation (Co-IP) with two-dimensional liquid chromatography coupled with tandem mass spectrometry (2D-LC-MS/MS), our results revealed that CD151 played the crucial role in the progression of HCC through formation of tetraspanin CD151 network with molecular partners, such as Mortalin, integrins α6β1 and c-Met 9. Moreover, the role of CD151 was influenced by its partner, and disassociation of tetraspanin CD151/integrins α6β1 using targeted monoclonal antibody (generated in our group) could inhibit the mobility and invasion of HCC cells *in vitro* 10, 11. Therefore, to explore the mechanism that keeps the structural stability of tetraspanin CD151 network is of significance for further disclosing the role of CD151 in HCC cells.

HCC is one of the most malignancies and ranks as the third most common cause of cancer-related mortality in the world 12, 13. The dismal outcome mainly contributes to highly metastatic ability of HCC cells 14. Therefore, it is considerable significance to disclose the role and molecular mechanism of key gene in the progression of HCC. Mortalin (also named as HSPA9, mthsp70, PBP74, Grp75) is a highly conserved molecular chaperone in the heat shock protein (HSP) 70 family 15. Mortalin is expressed in all cell types and tissues and performs cytoprotective functions by unfolding of proteins outside mitochondria and unidirectional translocation across mitochondrial membranes, and completes import by acting as an ATP-driven motor. Its function is induced by stress stimulus, such as ionizing radiation, glucose deprivation, calcium ionophore, ozone and hyperthyroidism 16. Recent studies reported that many of the human transformed cells and tumor cells including HCCs, had a high level of Mortalin expression 17. Overexpression of Mortalin was inclined to support the malignancy of carcinoma cells. However, the role and mechanism of Mortalin in HCC remain largely elusive.

In present study, we investigate the expression of Mortalin in HCCs, then analyze the interaction between Mortalin and CD151 in HCC cells. Finally, we evaluate the clinical implication of Mortalin and CD151 in HCCs.

## Materials and Methods

### Cell lines and culture

Four HCC cell lines Hep3B, HepG2 (purchased from ATCC), MHCC97L and HCCLM3 (established and preserved in Liver Cancer Institute of Fudan University) were used in present study and routinely raised 2, 18.

### Immunofluorescent staining, Western blot and quantitative real-time polymerase chain reaction (*q*RT-PCR)

Immunofluorescent staining was used to detect the location of CD151 and Mortalin in Hep3B, HepG2, MHCC97L and HCCLM3 cells as described previously 7. Rabbit anti-human Mortalin monocolonal antibody (Cell Signaling Technology) and mouse anti-human CD151 monocolonal antibody (established by our team) were used at 1:200 dilutions. Western blot analysis was used to examine the expression of CD151 and Mortalin in HCC cells as described previously 7. Rabbit anti-human Mortalin monocolonal antibody (Cell Signaling Technology) and mouse anti-human CD151 monocolonal antibody (generated in our team) were used at 1:1000 dilutions. Goat anti-human β-actin antibody (1:2000) was used as control. Mortalin and CD151 mRNA expression in HCC cells were examined by *q*RT-PCR as described previously. For the PCR amplification, primers were used for Mortalin: 5-CCCCAAGTAAAGCTGTCAATCCT-3, 5-GACCATCAGCGGCAGTAGAGAAT-3; CD151: 5'-ACTTCATCCTGCTCCTCATCAT-3', 5'-TCCGTGTTCAGCTGCTGGTA-3'; GAPDH: 5-GGGGCTCTCCAGAACATCATCC-3, 5-ACGCCTGCTTCACCACCTTCTT-3. GAPDH was used as control.

### Transfection and interference

Stable transfectant pGCSIL-GFP-shRNA-CD151 in HCC cells were constructed as previously described 8. The lentiviral-mediated pGCSIL-GFP-shRNA-mortalin was constructed as previously described (Shanghai Genechem Company Ltd., Shanghai, China). The shRNA targeting sequence for mortalin was identified high efficiency and used in subsequent experiment: 5'- ACATTGTGAAGGAGTTCAA-3'.

### Immunoprecipitation assay

The protein interaction between Mortalin and CD151 was investigated in HCCLM3 cells using co-immunoprecipitation as described in our previous study 11. The primary antibodies rabbit anti-human Mortalin monocolonal antibody (Cell Signaling Technology) and mouse anti-human CD151 monoclonal antibody (produced by our team) were used at 1:1000 dilutions. IgG was used as control.

### Wound healing and migration assay

A wound-healing assay was used to assay the role of CD151 and Mortalin in cell migration as described. Migration assay was performed as described elsewhere 10.

### Patients and follow-up

187 cases of HCC tumor specimens were collected as previously described 2. Detailed clinicopathological characteristics were listed in **Table 1**. Follow up procedures were consistent with our previous study 8.

### Tissue microarray (TMA) and immunohistochemistry

TMA was constructed as described previously 2, 19. The monoclonal mouse anti-human CD151 (1:200, established by our team) and rabbit anti-human Mortalin antibody (1:100, Cell Signaling Technology) antibodies were used to detect the expression of CD151 and Mortalin, respectively, based on a two-step protocol as previously described 8. The density of positive staining of CD151 and Mortalin was measured as described elsewhere 8, 20.

### Immunofluorescence

The immunofluorescence was performed according to our previous study 2.

### Statistical analysis

Statistical analysis was done with SPSS 16.0 software (SPSS, Chicago, IL). Student's t test was used for comparisons between groups. Categorical data were analyzed by the chi-square tests. Kaplan-Meier method and the log-rank test were used to assay the survival rates. *p*<0.05 was set as significant difference.

## Results

### High Mortalin expression play an important role in the progression of HCC cells

**Figure 1:**
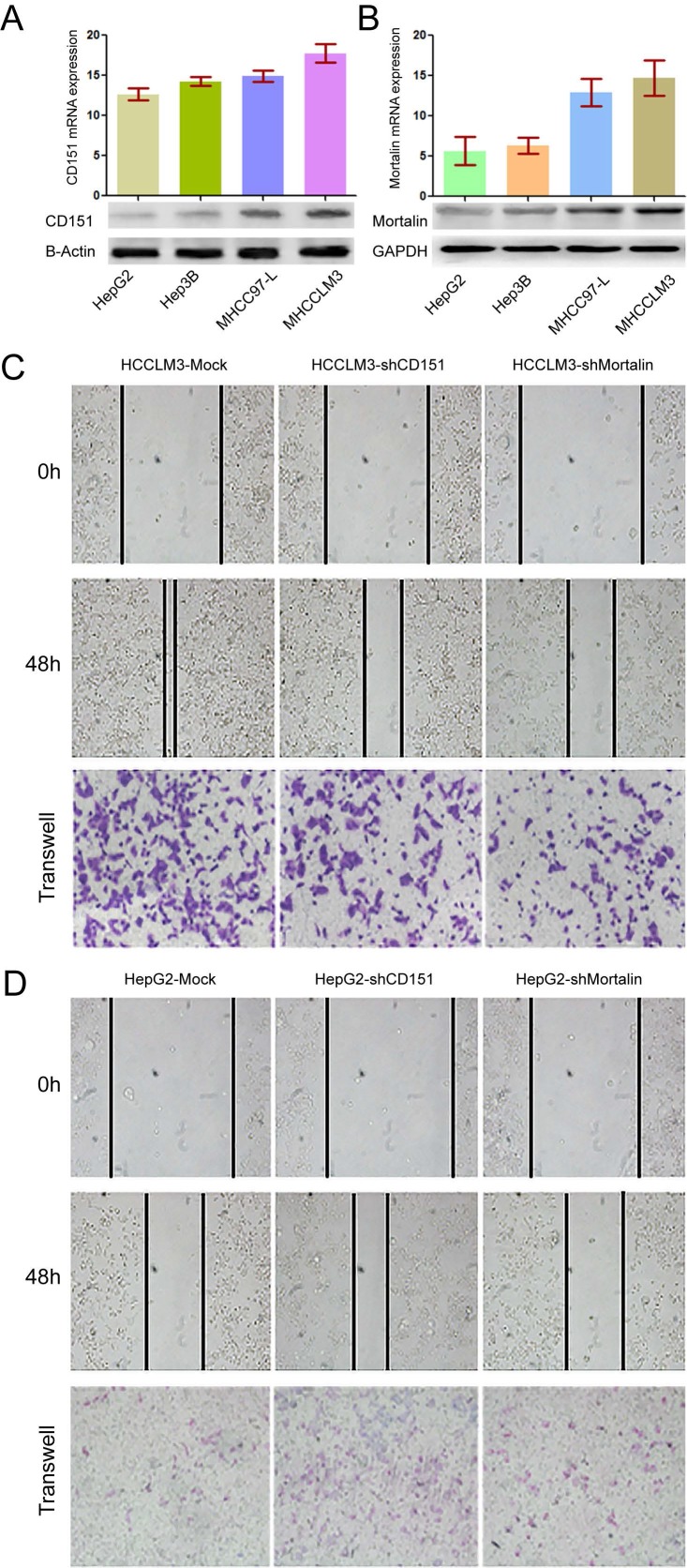
** Expression of CD151/Mortalin in HCCs and functional analysis. A.** Expression of CD151 protein and mRNA in HCC cells with different metastatic potential. **B.** Expression of Mortalin protein and mRNA in HCC cells with different metastatic potential. **C.** Knockdown of CD151 or Mortalin expression in HCCLM3 cells resulted in a decreased migration and invasion. **D.** Upregulation of CD151 or Mortalin expression in HepG2 cells resulted in an increased migration and invasion.

Previous study showed HCC patients with high CD151 expression were inclined to have poor prognosis 2. Here, we investigated the relationship between Mortalin and CD151 in HCC cells. Consistent with the results from our previous study 2, high metastatic HCCLM3 cells expressed the highest expression of CD151 at the protein and mRNA levels, while non-metastatic HepG2 presented the weak expression of CD151 (**Figure 1A**, *p*<0.05). In present study, the results showed that Mortalin expression in HCCLM3 cells was significantly higher than that in MHCC97-L (*p*<0.05) and HepG2 (*p*<0.01) cells at the protein and mRNA levels (**Figure 1B**). Next, we modified the expression of CD151 and/or Mortalin in HCC cells and assayed their roles in the invasion and migration of HCC cells. Expectedly, knockdown of CD151 expression in HCCLM3 cells reduced in a decreased migration and invasion (**Figure 1C**, *p*<0.05). Meanwhile, inhibition of Mortalin expression in HCCLM3 cells resulted in an impaired ability of migration and invasion as well (**Figure 1C,** *p*<0.05). Upregulation of CD151 in HepG2 cells resulted in an increased migration and invasion. When Mortalin expression in HepG2-CD151 cells were downregulated, their ability of migration and invasion were also impaired (**Figure 1D**, *p*<0.05). The results indicate that Mortalin play an important role in the progression of HCCs.

### Mortalin forms a complex with CD151 in HCC cells

**Figure 2:**
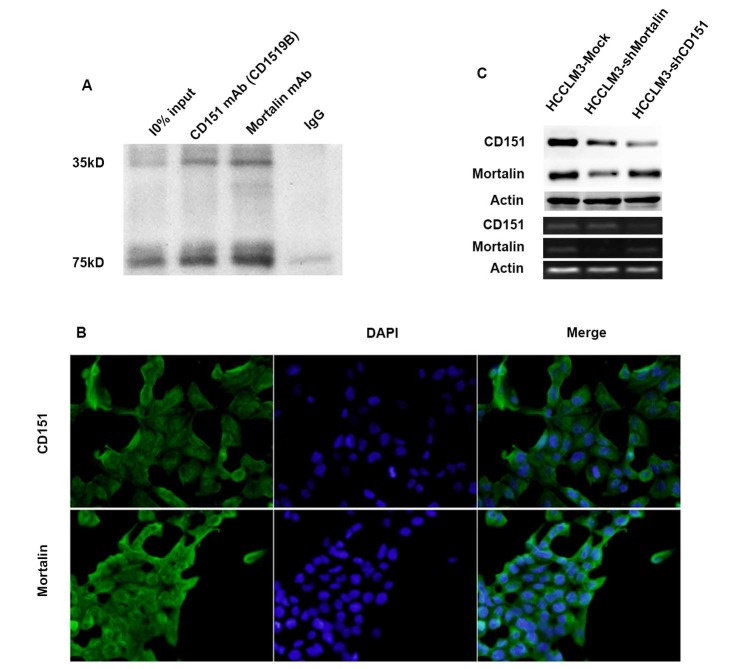
** Mortalin formed a complex with CD151 in HCCs. A.** Co-IP assay showed Mortalin formed a complex with CD151 in HCC cells. **B.** Immunofluorescence showed colocalization of CD151 and Mortalin in HCCLM3 cells (original magnification, ×400). **C.** Inhibition of Mortalin in HCC cells induced moderately decreased expression of CD151 protein, rather than CD151 mRNA. Downregulation of CD151 did not influence in the expression of Mortalin at the level of protein and mRNA.

Previously, we used the combination co-IP with 2D-LC-MS/MS to show that Mortalin may be one of partners for CD151 in HCCLM3 cells 9. Here, we further examined the relation between Mortalin and CD151 in HCCM3 cells expressing high level of CD151 protein using co-IP. The result revealed that Mortalin forms a complex with CD151 in HCCLM3 cells (**Figure 2A**). Next, we performed the immunofluorescence assay to investigate the location of CD151 and Mortalin in HCC cells, and found that Mortalin was localized on the plasma in HCC cells, while CD151 in plasma and membrane (**Figure 2B**). Then, we modified the expression of CD151 and/or Mortalin in HCC cells and investigated their interaction. Interestingly, inhibition of Mortalin in HCC cells induced moderately decreased expression of CD151 protein, rather than CD151 mRNA. While downregulation of CD151 did not influence in the expression of Mortalin at the level of protein and mRNA (**Figure 2C**). Given the cytoprotective role of heat shock protein family, we suggest that Mortalin could protect CD151 protein from degradation.

### CD151 involved in the progression of HCCs in a Mortalin-dependent manner

**Figure 3:**
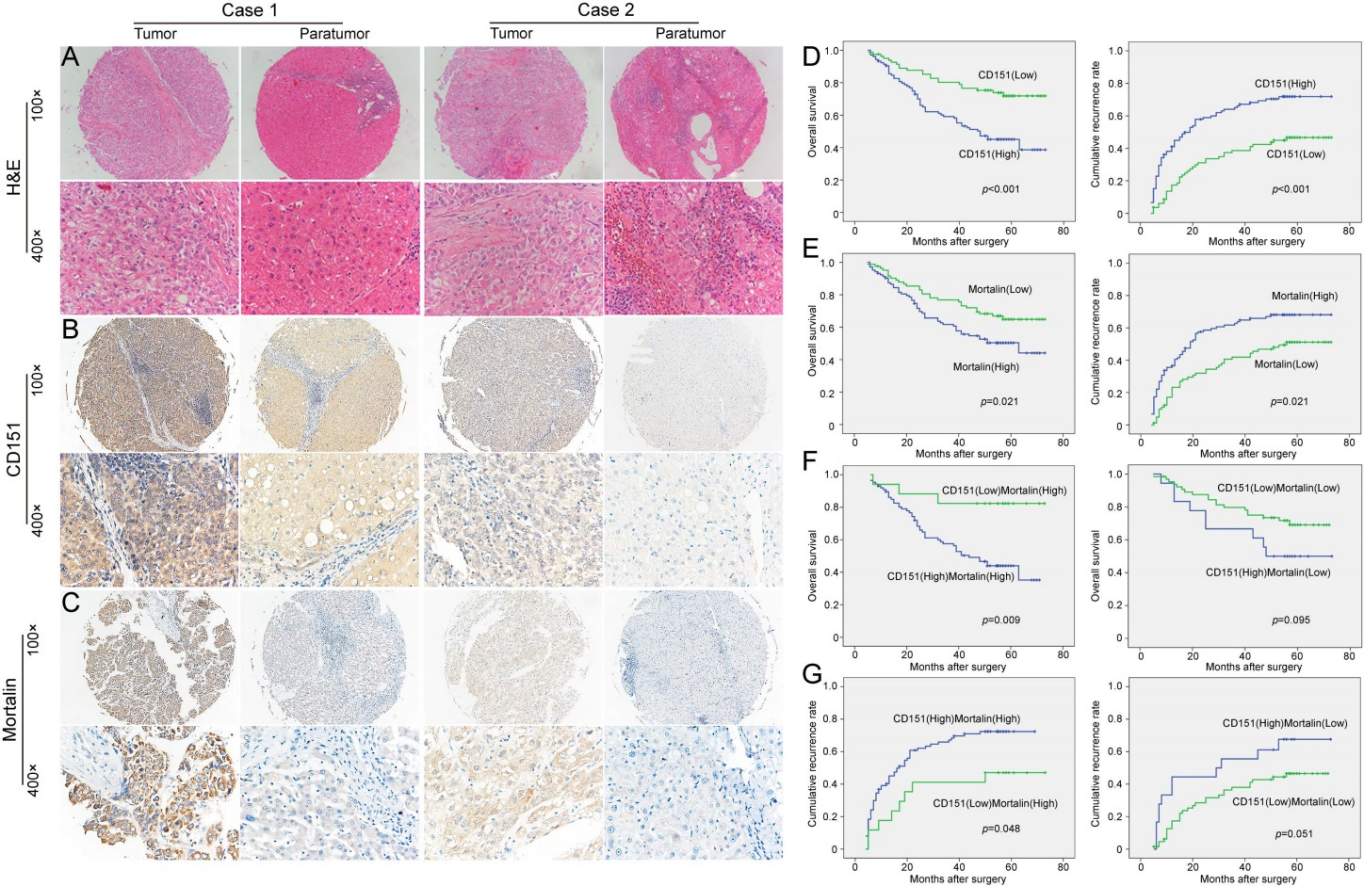
** The expression and prognostic implication of CD151/Mortalin in HCC. A.** Diagnosis by Hematoxylin and eosin (H&E) staining **B.** Expression of CD151 in tumor tissues was stronger than that in paratumoral tissues. Moreover, the expression of CD151 in different tumor sample had evidently variation. **C.** Tumor tissues had higher expression of Mortalin than that of paratumoral tissues. Moreover, the expression of Mortalin in tumor tissues had evidently variation. **D.** Prognostic implication of CD151 expression in HCC patients **E.** Prognostic implication of Mortalin expression in HCC patients **F and G.** Among Mortalinhigh subgroup of HCC patients, those with CD151high had shorter OS and higher cumulative recurrence rates than those with CD151low. However, in Mortalinlow subgroup of HCC patients, CD151 expression did not exert on the prognosis.

Here, we further investigated the implication of the CD151/Mortalin expression in a clinical setting including 187 cases of HCCs. After identification of primary HCC using hematoxylin-eosin staining (**Figure 3A**), we stained for CD151 and Mortalin in tissue microarray (TMA) slides using immunohistochemistry. Immunoreactivity of CD151 protein was located on the cell membranes and its intensity in tumor tissues was stronger than that in paratumoral tissues (**Figure 3B**). Positive Mortalin was expressed in the cytoplasm in a diffuse or granular pattern and its intensity in tumor samples was much stronger than that in paratumoral samples (**Figure 3C**). Next, we classified the whole cohort of patients into Mortalin^high^ and Mortalin^low^ subgroup according to Mortalin expression and analyzed the relationship between Mortalin expression and clinical characteristics. Our results revealed that expression of Mortalin was positively correlated with microvascular invasion (*p*=0.017) and tumor diameter (*p*=0.001). However, other clinical characteristics, including age, sex, serum alpha fetoprotein, Child-Pugh score, tumor number, tumor capsulation and differentiation were not directly related to the expression of Mortalin. As described in our previous study, high level of CD151 was significantly related to tumor size (>5cm) (*p*=0.007) and vascular invasion (*p*=0.003) (**Table 1**).

Then, we investigated the clinical implication of Mortalin/CD151 in the prognosis of HCC patients. In line with our previous results, present study also showed that CD151 expression was inversely related to the poor prognosis of HCC (**Figure 3D**). The OS rate of the Mortalin^low^ group was higher than those in the Mortalin^high^ group (66.3% versus 51%). The cumulative recurrence rates of the Mortalin^high^ group was significantly higher than that of the low level of Mortalin group (66.3% versus 49.4%, **Figure 3E**). Given the role of Mortalin in the stability of CD151 protein, we investigated the clinical implication of CD151 expression in the subgroup with different level of Mortalin. Interestingly, among Mortalin^high^ subgroup of HCC patients, HCC patients with CD151^high^ had shorter OS and higher cumulative recurrence rates than those with CD151^low^ (**Figure 3F and G**). However, in Mortalin^low^ subgroup of HCC patients, CD151 expression did not exert on the prognosis (**Figure 3F and G**). These data also support the notion that Mortalin probably prevent from degradation of CD151 protein and involve in the progression of HCCs. A Cox proportional hazards model showed CD151 was an independent prognostic indicator for OS (*p*=0.011) and cumulative recurrence (*p*=0.002) (**Table 2 and 3**). While Mortalin was an independent prognostic indicator for cumulative recurrence (*p*=0.046) (**Table 3**). When CD151/Mortalin was adopted as a covariate, we found that CD151/Mortalin was also an independent prognostic predictor for cumulative recurrence (*p*=0.021) (**Table 3**).

## Discussion

In present study, our results revealed that high metastatic HCC cells tend to express high level of Mortalin. Interference of Mortalin significantly inhibited the invasion and migration of HCC cells. Clinically, HCC patients with Mortalin overexpression had poor prognosis. Therefore, we conclude that Mortalin does play an important role in in the progression of HCC.

A more interesting result from our study is that Mortalin could form a complex with CD151 and prevent from destabilization of CD151-depedent TEM and involve in the progression of HCC. TEM was considered as a function unit for tetraspanin family, and its stability is a requisite for the role of tetraspanin CD151 related to the invasion and metastasis in malignant tumors, including HCC 2. In our study, high metastatic HCC cells express high level of Mortalin and CD151. Mortalin formed a complex with CD151 in HCC cells. Importantly, down-regulation of Mortalin induced a moderate decreased CD151 protein, but not CD151 mRNA, while inhibition of CD151 did not influence the expression of Mortalin. Moreover, upregulation of CD151 expression in HCC cells partially restored the ability of invasion and migration of HCC cells induced by interference of Mortalin. More importantly, HCC patients with CD151 overexpression had poor prognosis, to a large extent, depending on high Mortalin expression in tumor tissues. These data support our notion that Mortalin stabilize CD151-depedent TEM and involve in the progression of HCC. Mortalin exists in multiple subcellular sites of cell, including the mitochondrion, plasma membrane, endoplasmic reticulum, cytosol, and nucleus. It may serve as safeguards to maintain homeostasis and integrity of protein interactions and play a vital role in multiple processes of cell, such as stress response, intracellular trafficking, cell proliferation, and differentiation 21. Recent studies have focused on the role of Mortalin in carcinogenesis and tumor progression. Mortalin could efficiently protect cancer cells from endogenous and exogenous oxidative stress 22. Mortalin also inactivated tumor suppressor protein p53 functions and activated telomerase and heterogeneous ribonucleoprotein K (hnRNP-K) proteins, thus promoting carcinogenesis and tumor metastasis 23. Starenki D *et al* 24 reported that mortalin was upregulated in human medullary thyroid carcinoma (MTC) tissues and its depletion robustly induces cell death and growth arrest by inducing transient extracellular signal-regulated kinase (MEK/ERK) activation and altering mitochondrial bioenergetics. Chen J *et al* 17 also found that the overexpression of Mortalin was correlated with the metastatic phenotype of HCC cells and promoted the progression by induction of the EMT. In our serial studies, CD151 was validated as a key gene related to the invasiveness and metastasis of HCC. CD151 could form a complex with integrin α6β1 and c-Met and involved in several pathological processes, such invasiveness, neoangiogenesis and EMT 2, 8. The function of CD151 depends on the stability of TEM. Our study also confirmed that CD151 antibody targeting the CD151-integrin α6β1 binding domain could disassociate the TEM and inhibit the function of CD151 9. Therefore, as one of the molecular chaperones of CD151, Mortalin efficiently stabilized the CD151-depended TEM. Certainly, our study has some limitation as well. For example, the binding site of CD151/Mortalin need to be uncovered.

## Figures and Tables

**Table 1:** Correlation between CD151/Mortalin expression and clinicopathological features in 187 hepatocellular carcinoma patients

Variables	CD151 staining		*p* value	Mortalin staining		*p* value
	High	Low		High	Low	
**Sex (female vs. male)**						
Male	91	69	0.627	88	72	0.680
Female	14	13		16	11	
**Age(years)**						
≥53	53	36	0.372	51	38	0.658
<53	52	46		53	45	
**HBsAg**						
Positive	87	67	0.838	88	66	0.364
Negative	18	15		16	17	
**Child-Pugh score**						
A	102	80	0.607^*^	101	81	0.827^*^
B	3	2		3	2	
**ALT(U/ml)**						
≥75	89	75	0.166	88	76	0.150
<75	16	7		16	7	
**Serum AFP (ng/ml)**						
≥20	64	60	0.079	68	56	0.764
<20	41	22		36	27	
**GGT(U/ml)**						
≥75	35	20	0.183	32	23	0.648
<75	70	62		72	60	
**Cirrhosis**						
Yes	96	73	0.580	95	74	0.614
No	9	9		9	9	
**Diameter(cm)**						
≥5	54	26	0.007	56	24	0.001
<5	51	56		48	59	
**Tumor number**						
Multiple	19	11	0.387	16	14	0.784
Solitary	86	71		88	69	
**Microvascular invasion (yes vs no)**						
Yes	40	15	0.003	38	17	0.017
No	65	67		66	66	
**Tumor Capsulation**						
Yes	48	42	0.455	48	42	0.545
None	57	40		56	41	
**Tumor differentiation**						
III/IV	76	60	0.904	30	21	0.589
I/II	29	22		74	62	

Abbreviations: HBsAg, Surface of antigen of Hepatitis B virus; ALT, Alanine transaminase; AFP, alpha-fetoprotein; GGT, Gamma-Glutamyltransferase. *Fisher's Exact Test

**Table 2:** Univariate and multivariate analyses of factors associated with overall survival.

Factors	Univariate, *p*	Multivariate			
		HR	95%Cl		*p* Value
Sex (female vs. male)	0.203				NA
Age (years) (≥53 vs. <53)	0.217				NA
HBsAg (positive vs. negative)	0.087				NA
Child-Pugh classification (A vs. B)	0.149				NA
Serum ALT, U/L (≥75 vs. <75)	0.260				NA
Serum AFP, ng/L (≥20 vs. <20)	0.298				NA
Liver cirrhosis (yes vs. no)	0.593				NA
Tumor size (diameter, cm) (<5 vs. ≥5)	<0.001	0.510	0.317-0.823		0.006
Tumor number (multiple vs. single)	0.048				NS
Tumor capsulation (yes vs. no)	0.168				NA
Tumor differentiation (III/IV vs. I/II.)	0.084				NA
Microvascular invasion (yes vs. no)	<0.001	2.231	1.383-3.597		0.001
TNM stage (I/II vs. III/IV)	0.001				NA
CD151 expression (high vs. low)	<0.001	0.515	0.310-0.858		0.011
Mortalin expression (high vs. low)	0.023				NS
CD151/Mortalin expression (low vs. high)	<0.001				NS
					

Abbreviation: 95% CI: 95% confidence interval; AFP, alpha-fetoprotein; TNM, tumor-node-metastasis; HBsAg, hepatitis B surface antigen; HR, hazard ratio; NA, not adopted; NS, not significant; OS, overall survival. Cox proportional hazards regression model.

**Table 3:** Univariate and multivariate analyses of factors associated with cumulative recurrence rate.

Factors	Univariate, *p*	Multivariate		*p* value
		HR	95%Cl	
Sex (female vs. male)	0.245			NA
Age (years) (≥53 vs. <53)	0.285			NA
HBsAg (positive vs. negative)	0.483			NA
Child-Pugh classification (A vs. B)	0.879			NA
Serum ALT, U/L (≥75 vs. <75)	0.838			NA
Serum AFP, ng/L (≥20 vs. <20)	0.099			NA
Liver cirrhosis (yes vs. no)	0.176			NA
Tumor size (diameter, cm) (≥5 vs. <5)	<0.001	0.549	0.368-0.819	0.003
Tumor number (multiple vs. single)	0.005			NS
Tumor Capsulation (yes vs. no)	0.051			NA
Tumor differentiation (III/IV vs. I/II.)	0.078			NA
Microvascular invasion (yes vs. no)	<0.001	1.736	1.140-2.644	0.010
TNM stage (I/II vs. III/IV)	0.002			NA
CD151 expression (low vs. high)	<0.001	0.532	0.354-0.799	0.002
Mortalin expression (low vs. high)	0.003	0.664	0.444-0.992	0.046
CD151/Mortalin expression (low vs. high)	<0.001	0.626	0.421-0.932	0.021

Abbreviation: 95% CI, 95% confidence interval; AFP, alpha-fetoprotein; TNM, tumor-node-metastasis; HBsAg, hepatitis B surface antigen; HR, hazard ratio; NA, not adopted; NS, not significant; OS, overall survival. Cox proportional hazards regression model.

## Abbreviations

HCC: hepatocellular carcinoma
AFP: α-fetoprotein
PI3K: phosphatidylinositol 3-kinase
Co-IP: co-immunoprecipitation
MS: mass spectrometry
TMA: tissue microarray
TNM: tumor node metastasis
shRNA: short hairpin RNA
qRT-PCR: quantitative real-time polymerase chain reaction
2D-LC-MS/MS: two-dimensional liquid chromatography coupled with tandem mass spectrometry
DMEM: Dulbecco's modified Eagle medium
FBS: fetal bovine serum
PBS: phosphate buffer solution
DAPI: 4',6-Diamidino-2-phenylindole
BSA: bovine serum albumin
SDS: sodium dodecyl sulfate
OS: overall survival
